# Identifying perceived barriers and enablers of healthy eating in college students in Hawai’i: a qualitative study using focus groups

**DOI:** 10.1186/s40795-019-0280-0

**Published:** 2019-02-22

**Authors:** Lucia Amore, Opal Vanessa Buchthal, Jinan C. Banna

**Affiliations:** 1 Department of Human Nutrition, Food, and Animal Sciences, College of Tropical Agriculture and Human Resources, Agricultural Sciences 216, University of Hawaii at Manoa, 1955 East-West Road, Honolulu, HI 96822 USA; 2 Office of Public Health Studies, University of Hawaii at Manoa, 1960 East-West Road, Honolulu, HI 96822 USA; 3 Department of Human Nutrition, Food, and Animal Sciences, College of Tropical Agriculture and Human Resources, Agricultural Sciences 216, University of Hawaii at Manoa, 1955 East-West Road, Honolulu, HI 96822 USA

**Keywords:** College students, Focus groups, Qualitative research, Healthy diet, Hawai’i, Socio-ecological model

## Abstract

**Background:**

To design effective nutrition education interventions for college students, research is needed to determine the factors influencing food choices. The purpose of this study was to identify perceived barriers and enablers of healthy eating in college students ages 18–24 at the University of Hawai’i at Mānoa.

**Methods:**

Prior to conducting focus groups, an interview guide was developed based on a literature review of relevant studies. The interview guide was successfully tested in the first focus group and used in the rest of the focus groups. Eleven focus groups with group sizes of two to six were conducted (*n* = 44). Focus groups were audio-recorded and transcribed. Transcripts were coded in NVIVO11 using content analysis, and additional codes were added to the codebook based on emergent ideas from the transcripts. After completion of the final codebook, transcripts were recoded with the new codebook. Final code counts were used to identify overarching ideas based on the socio-ecological model of health, consisting of four levels of influence: individual (intrapersonal), social environmental (interpersonal), physical environmental (community settings), and macrosystem (societal).

**Results:**

The largest barriers according to level of influence in the socio-ecological model were nutrition knowledge deficit (individual), peer pressure (social environmental), unsupportive institutional environment (physical environmental), and cost (macrosystem). The largest enablers were nutrition knowledge (individual), parental influence (social environmental), an institutional environment with consistent healthy offerings (physical environmental), and social media (macrosystem). Some factors served as barriers for some participants and enablers for others, such as parental influence.

**Conclusion:**

Factors such as individual knowledge and parental support were cited as having a positive influence in promoting healthy eating, while factors such as the cost of living and food availability at college serve as barriers even for motivated students. Results from this study identify potential areas of intervention, such as improving nutrition knowledge (individual), offering more healthy options (physical environmental), or reducing cost of food (macrosystem). However, more research is needed to identify which level of intervention may be most effective in changing food habits, and which barriers or enablers are deciding factors in determining this population’s food choices.

**Electronic supplementary material:**

The online version of this article (10.1186/s40795-019-0280-0) contains supplementary material, which is available to authorized users.

## Background

College students have poor nutritional habits, with fruit and vegetable consumption below the recommended five servings a day and frequent fast food or fried food consumption [1, 2]. The college environment has been termed an “obesogenic environment” due to high access to low nutrient, energy dense foods and the high-calorie environment of the university dorm [3]. College students may not immediately realize the impact of their poor nutritional habits because college students have high energy metabolism as they reach peak lean body mass (a significant contributor to resting energy expenditure) around the time of college years [4]. With high energy expenditure, calories consumed in the college years are greater than at other points in life.

Eating behaviors of college students may carry over to later life, as the college years are a critical period in habit formation [5]. For this reason, the college years are also a potential period of intervention. The college years present an opportunity to acquire healthy habits as students enter emerging adulthood, in which change occurs more frequently than at any other point in life [6]. While the college years are a potential period of intervention, recent research points to the lack of nutrition education for college students, particularly in healthy weight management [7].

To design effective nutrition education interventions for college students, research is needed to determine the factors influencing college students’ food choices. Previous research studies using focus groups have identified several determinants of eating behaviors in college students, such as taste preference, availability and accessibility of foods, cost, and other college life factors such as the characteristics of the university, student societies, and exams [8–11]. Several of these studies have referenced the socio-ecological model, which identifies influences at the individual, social environmental, physical environmental, and macrosystem levels and is used in health promotion to better understand not only the individual but the unique environment in which he/she lives [12]. Previous studies examining the barriers and enablers to healthy eating in college students have been conducted in the continental US, Europe, Australia, and New Zealand, but there have been no such studies performed in Hawai’i. To address the needs of this population, the objective of this study was to identify barriers and enablers of healthy eating in college students in Hawai’i.

## Methods

The University of Hawai’i at Mānoa (UHM) Institutional Review Board approved the study.

### Participants

Participants were full-time students in the age range of 18–24 at UHM, the largest college in Hawai’i. Eleven focus groups containing two to six participants were conducted (*n* = 44). Students were recruited via email, class listservs, flyers, and word of mouth.

### Question guide

A question guide was developed based on findings of similar studies in young adults (Additional file 1) [9, 10]. The question guide was tested in one focus group to confirm that it would yield responses relevant to identifying barriers and enablers of healthy eating.

### Initial codebook development

Prior to conducting focus groups, one researcher (LA) developed a preliminary codebook based on previous studies. Barriers and enablers of healthy eating identified in the codebook were organized based on the socioecological model [13], with codes in four levels of influence: individual, social environmental, physical environmental, and macrosystem. For example, codes in the “individual” category included “knowledge,” “behaviors,” and “attitudes and beliefs.” Codes in the “social environmental” category included “peer influence” and “parental influence,” while codes in the “physical environmental” category included “institutional environment,” “living situation,” and “facilities.” Lastly, codes related to the macrosystem included “cost of food” and “social media.” The codebook was expanded as focus group transcripts were analyzed, and two other researchers (OB and JB) reviewed the codes. These procedures were conducted to ensure validity of findings [14].

### Focus groups

Focus groups conducted by a trained moderator (LA) were held on the UHM campus. On the day of the focus group, all students read and signed the consent form and completed a demographic survey. The focus groups typically lasted approximately one hour and were audio-recorded. Students received a $10 gift card to a local grocery store as an incentive.

Audio recordings were subsequently transcribed verbatim. Focus groups were held until the point of data saturation, or the point at which after analysis, no more themes emerged [15].

### Data analysis and final codebook development

Data were analyzed using NVivo software package version 11 (QSR International Inc., Burlington, MA, USA) using content analysis [16]. Beginning with the initial codebook based on the literature and organized by levels in the social ecological model, LA coded the transcripts after each focus group was conducted. Initial coding was done according to the levels of influence identified in the socio-ecological model: individual (intrapersonal), social environmental (interpersonal), physical environmental (community settings) and macrosystem (societal).

This coding was then reviewed and discussed amongst the researchers, and most common ideas within each of the four levels were identified. For example, nutrition knowledge deficit as a theme emerged at the individual level, and the researchers created a new code at the individual level. As another example, peer pressure was identified as a common idea at the social environmental level and a new code was created. These new codes were added after reviewing the quotations and frequency using code counts. As additional codes were identified, such as lack of facilities, location, or thriftiness, the codebook was expanded. Operational definitions for codes were developed, and codes were discussed amongst all researchers. The key barriers and enablers were determined by examining code counts and discussed among all researchers.

As additional focus groups were conducted, the codes were reviewed and new codes were added. These new codes were further explored in subsequent focus groups and used to re-code previous focus group transcripts.

The final codes were determined after re-coding by the most recent codebook and consensus by the three researchers, with confirmation in the final focus group. Because no new codes were identified in the final focus group, it was concluded that the point of data saturation had been reached.

## Results

The characteristics of students who participated in focus groups are displayed in Table 1. Student major varied, with 36% enrolled in biology or other biology-related programs and 14% enrolled in health-related programs. Over half of students were at normal weight, while the rest were underweight (14%) or overweight (18%). A large proportion identified as Asian (39%), a slightly smaller number identified as White (34%), and 20% were multiracial.

**Table 1:** Demographic characteristics of focus group participants at UHM (*n* = 44)

	Characteristic	Total (n[%])
Gender	Female	33 (75%)
	Male	11 (25%)
	Age (yrs) (mean ± SD)	19.4 ± 1.9
BMI Category	BMI (kg/m^2^) (mean ± SD)	22.7 ± 3.5
	Underweight (BMI < 18.5)	6 (14%)
	Normal (BMI 18.5–25)	26 (60%)
	Overweight (BMI 25–30)	8 (18%)
	Obese (BMI 30+)	1 (2%)
	No response	3 (7%)
Hispanic	Hispanic	8 (18%)
	Not Hispanic	36 (82%)
Race	Multiracial^a^	9 (20%)
	Asian (Chinese, Filipino, Japanese, Korean, Vietnamese)	17 (39%)
	White	15 (34%)
	Not reported	3 (7%)
Class Standing	Freshmen	18 (41%)
	Sophomore	6 (14%)
	Junior	8 (18%)
	Senior	7 (16%)
	5th year / grad student	1 (2%)
	No Response	4 (9%)
Major	Biological Sciences^b^	16 (36%)
	Health Focus^c^	6 (14%)
	Other	22 (50%)

^a^Multiracial: Participants who reported as ≥2 races

^b^Biological sciences: majors pertaining to biology and other related studies (microbiology, marine biology, etc..)

^c^Health focus was defined as majors pertaining to nutrition, medicine, and kinesiology

Barriers and enablers to healthy eating behaviors were identified at the four levels of the socio-ecological model. Themes are displayed in order of prominence by socio-ecological level in Tables 2 and 3, along with exemplifying quotations. Some barriers were also perceived as enablers, and thus appear in both tables. More barriers than enablers were identified.

**Table 2:** Key barriers to healthy eating in college students at UHM (n = 44)

	Barrier	Definition	Exemplifying Quotation
Individual^a^	Lack of Knowledge	Lack of knowledge of how to obtain or prepare food, lack of nutrition knowledge or inability to identify healthy foods	*“I think my biggest barrier is definitely not knowing what I’m doing. Just not knowing what to buy, not knowing what to cook to be healthy.”*
	Attitudes and Beliefs	Regarding healthy eating as undesirable	*“I feel like eating healthy is kind of a chore. It’s healthy but it doesn’t taste the best. It doesn’t make me happy, because you’re supposed to enjoy eating.”*
	Attitude and Beliefs: Prioritization	Regarding healthy eating as not a priority in relation to other factors	*“After a long day of school […] we worry more about convenience than health.”*
	Attitude and Beliefs: Procrastination	Regarding healthy eating as not urgent	*“Not until something affects them do they really realize like, ‘Oh I should probably change my eating habits’”*
	Attitude and Beliefs: Thriftiness	Believing resources should be used carefully and waste avoided	“*I think it’s hard for a lot of college students to eat healthy in the dining halls because it’s all-you-can-eat. You want to get your money’s worth.*”
	Behaviors	Performing peripheral behaviors that hinder healthy eating	*“You take a handful of [brand name snack] and go study. Studying is really boring and you’re not focusing well. Then you get another handful and then you go get another handful. Then you bring the box in your room [laughter]. And […] you’re basically unconsciously just eating them as you study and you don’t even recognize [how much] you’ve eaten.”*
	Taste Preference	Preference for the taste of foods perceived to be unhealthy	“*I’m not going to suffer through gross, I’m going to go eat pizza [laughs].”*
Social Environmental^b^	Peer Pressure	Pressure from peers to engage in unhealthy eating behaviors	*“I would never eat past 8, but when everyone’s hanging out, eating like chips and stuff, […] and it’s there and [you’re] like, “Oh okay, I’ll have like a few chips.” I’d never […] do that at home. So it’s the social part.”*
	Parental Influence	Parental influence that encourages unhealthy eating behaviors	*“Students may also change their eating habits on purpose because they were limited by their parents or guardians. Because it’s ‘Eat your vegetables,’ and now there’s nobody so there’s no restrictions.”*
Physical Environmental^c^	Institutional Environment	Aspects of the college environment that hinder healthy eating	*“There’s definitely ways to be healthy on campus, but there’s a lot of places here that have better, healthier options that are way more expensive.”*
	Living Situation	Negative impact of living situation on available food options	*“If I had an apartment with a kitchen I would be better off because I could just cook for myself.”*
	Lack of Facilities	Lack of food storage space or utensils that hinder healthy eating	“*All I had was a mini fridge and a microwave and my food suddenly became ramen and whatever was just I could order [online] so not really many fruits or veggies”.*
	Location	Lack of proximity to grocery stores or commute hindering healthy eating	*“Because a lot of us aren’t from here, we don’t have that access—Well, I mean, we have the bus, but it’s not like we can take so many groceries with us on the bus. It’s difficult for us to… wander away from campus to go buy what we need.”*
Macrosystem^d^	Cost	Negative impact of cost on healthy food options	*“I can get a lot more out of my money if I’m buying things like pastas, cereals, those kinds of fast filling foods. Rather than buying fresh fruit, vegetables, and meat [which are] expensive as well […] it’s much more cost effective.”*
	Lack of Education	Characteristics of the educational system that hinder healthy eating	*“In the education system, we’ve taken out so many things that are important, you know? When- when my parents- just older generations talk about school, they had a lot of life skills classes […] they had home-ec.”*

^a^Individual characteristics deterring participants from healthy eating, including psychosocial factors (attitudes and beliefs, knowledge, self-efficacy, preferences), behavioral factors (meal and snack habits and other food-related behaviors), and lifestyle factors (perceived barriers, cost, time, convenience)

^b^Interpersonal influences (including family, friends, peer networks, and other social groups that model and reinforce perceived norms) that encourage unhealthy eating behaviors

^c^Influences in the community setting which influence the accessibility and availability of foods, such as grocery stores, vending machines, cafeterias, etc. such that healthy eating is more difficult

^d^Influences pertaining to mass media, advertising, marketing, social norms, cultural norms, food production and distribution systems, local, state, and federal policies which influence food-related issues that serve as a barrier to healthy eating

**Table 3:** Key enablers of healthy eating in college students at UHM (*n* = 44)

	Enabler	Definition	Exemplifying Quote
Individual^a^	Knowledge	Knowledge or awareness of nutrition, understanding of dietary restrictions, and ability to identify healthy foods	*“Being aware really does help. I took nutrition and fitness last year. It honestly did change the way I ate a little bit because I just learned a lot about eating habits and what’s in food and things like that.”*
	Attitude and Beliefs	Perceptions that make healthy eating desirable	“*It’s not about the body for me, it’s about the energy. That’s how I look at it. Food is energy.”*
	Attitude and Beliefs: Prioritization	Belief that healthy eating is a priority in relation to other factors	*“I’m the most disorganized person ever. But [meal prep] is a priority in my life. So every Sunday I cook seven dinners and then snacks and then I freeze them.”*
	Attitude and Beliefs: Thriftiness	Belief that resources should be used carefully and waste avoided	*“A recipe makes a certain amount and you’re like “well I don’t want to waste this or it won’t fit in my fridge and to me being wasteful is really being part of being healthy.” Like being healthy to the planet.”*
	Dietary Restrictions	Having a health condition that requires a particular diet	*“last year, I had to go to the doctor a lot because I didn’t know what was going on with me. So I guess that’s why you have to be healthy [….] Even though you don’t want to do it, you still have to.”*
	Behaviors	Performing peripheral behaviors that foster healthy eating	*“I take time to meal prep and so I can eat healthy and it’s easier for me to choose a healthy snack.”*
Social Environmental^b^	Parental Influence	Parental influence on the home eating environment that encourages healthy eating behaviors	*“When it’s at home your parents monitor what you eat. Like, ‘No, you’re not going to eat half a pan of brownies.’”*
	Peer Support	Interpersonal support for healthy behavior change as a bonding/shared activity	*“[my best friend and I] go workout together, make dinner together. It was because that we had each other that we were like ok like ‘tonight we’re gonna do this it’s gonna be great’. You make it fun.”*
Physical Environmental^c^	Institutional Environment	Aspects of the college environment that foster healthy eating	*“One of the things I do like about UH though is the farmers market that they have. Where it has those fruits and vegetables. That’s at a really good price. So it’s almost like having a mini grocery store. So I appreciate the school giving us that much.”*
	Living Situation	Positive impact of living situation on available food options	*“Now I live off campus and I pack lunches every day, so I’m not buying the food [on campus] since there are the limited healthy options [….] I definitely see better eating habits now that I’m living off campus as opposed to living on campus.”*
Macrosystem^d^	Social Media	Positive impact of social media on eating habits	*“Social media now, too, is an enabler. Because there’s so many more like, vegan, vegetarian, like healthy food pages that you can find recipes on that are pretty make-able […] I think socially and society-wise, it’s being more promoted.”*
	Cost	Positive impact of cost on healthy food options	*“if we have a little more money […] then it might be easier for some students to figure out what food they want that’s more of a priority to them - which might be the more expensive healthier food.”*

^b^Individual characteristics that encourage healthy eating, including psychosocial factors (attitudes and beliefs, knowledge, self-efficacy, preferences), behavioral factors (meal and snack habits and other food-related behaviors), and lifestyle factors (perceived enablers, cost, time, convenience)

^b^Interpersonal influences (including family, friends, peer networks, and other social groups that model and reinforce perceived norms) that encourage healthy eating behaviors

^c^Influences in the community setting which influence the accessibility and availability of foods, such as grocery stores, vending machines, cafeterias, etc. such that healthy eating is easier

^d^Influences pertaining to mass media, advertising, marketing, social norms, cultural norms, food production and distribution systems, local, state, and federal policies which influence food-related issues that serve as an enabler of healthy eating

### Barrier – Individual

#### Lack of knowledge

Students reported a lack of nutritional knowledge, or nutrition misinformation as a reason for unhealthy eating. One participants stated, *“Some people are just unaware of what our body needs.”*

#### Attitudes and beliefs

Some individuals viewed healthy eating as undesirable, which served as a barrier. There were multiple reasons for this, such as the perception that healthy foods do not taste good or are not as satisfying as unhealthy foods. Some students considered healthy eating to be an obligation compared to eating junk food: *“‘why are you telling me to eat this; do I need to eat [vegetables]?’ It kinda becomes an obligation, versus junk food, where it’s […. ]freedom.”*

#### Attitudes and beliefs: Prioritization

For individuals, lack of prioritization of healthy eating in relation to school or other activities served as a barrier to healthy eating. Selecting healthy foods was perceived to detract from students’ ability to focus on school responsibilities because *“You only have so much mental energy.”* Participants usually reported prioritizing schoolwork over healthy eating.

#### Attitudes and beliefs: Procrastination

Some participants did not feel urgency in making healthy food choices. Participants reported delaying healthy choices until experiencing negative consequences. When asked why healthy eating was less of a priority, one said “*[A poor diet is] just for today. We don’t think of it more as ‘I’m running out of time.’ Or like, ‘I have all the time in the world to fix that tomorrow.’”*

#### Attitudes and beliefs: Thriftiness

Thriftiness, or the practice of using resources carefully and avoiding waste, emerged as a consideration in unhealthy eating habits, like overeating. Students reported seeking to maximize their money’s worth at buffets on campus (at UHM, the cafeteria offerings for students are served as “all-you-can-eat”). Thriftiness functioned as a barrier when students sought to avoid throwing away food by eating beyond satiety: “*You don’t want to throw the rest of it away cause you can’t finish it and then that’s wasting food when there’s people who don’t have food to eat or things like that.”*

#### Behaviors

Eating while bored or stressed was cited as a common barrier to healthy eating. Eating while distracted fostered overeating and mindless snacking without attention to quantity consumed. Students also reported not paying attention to what they were eating while stressed, especially during exam week. “*When [I’m] groggy, just […] give me anything. Anything is fine as long as I’m eating, but usually I end up turning to like some ice cream or something in the middle of the night when I’m stress eating, especially during finals week.”*

#### Taste preference

Other examples pertaining to food preference came from a preference for unhealthy food because of the taste compared to perceived healthy foods. Some participants had a preference for less healthy options because participants deemed unhealthy options more flavorful and desirable: “*If a salad was so good, I would buy it every day but for me I don’t think that salads are so delicious. I think the taste is what’s kind of blocking me from actually going out and eating healthy.”*

### Barrier – Social environmental

#### Peer pressure

Several students recalled instances of peer pressure in which personal inclinations were suppressed due to the influence of peers. For example, a participant reported a tendency to eat what her friends were eating: “*I’m following my friends. And they don’t really want to eat healthy.”*

Aside from peers, some participants reported gaining weight when going on regular dates with their significant other. Some of the female participants termed this the “*boyfriend effect*” and noticed a weight change from the time before they began dating their boyfriend to the time the focus group was conducted. Most participants attributed this to going out to eat more than they normally would on their own.

#### Parental influence

Parental influence and control over food environments during childhood and adolescence was important in determining eating habits in college. Too much parental control served as a barrier by making prohibited foods, which were usually less nutritious foods, more desirable once students could access them freely in the college environment. Lack of parental nutrition knowledge also impacted students because students grew up with a nutrition knowledge deficit: “*I know my family never really ate [what’s] healthy, so I never really grew up learning what I should buy to make [healthy foods I like].”*

### Barrier – Physical environmental

#### Institutional environment

While some students stated that there are desirably healthy foods on campus, they said that with the caveat that the cost is undesirable. With regard to the cafeteria food, students discussed that healthy food options are available and accessible, but also mentioned that *“we don’t have much variety… it’s the same things everyday, too.”* Students felt as if the healthier options, specifically vegetarian options, at the cafeteria were limited in that they were repetitive, had the appearance of *“leftover vegetables”,* had an undesirable taste*,* or were not a complete meal option: *“that’s not a dinner; that’s a side dish.”*

#### Living situation

“*Dorming*” was mentioned as a barrier to healthy eating behaviors due to lack of kitchen availability. A student who moved from the dorms to an off-campus location felt as if “*dorming*” resulted in limited healthy options: *“Now I live off campus and I pack lunches every day, so I’m not buying the food [on campus] since there are the limited healthy options [….] I definitely see better eating habits now that I’m living off campus as opposed to living on campus.”*

#### Lack of facilities

Lack of cooking facilities was reported as a hindrance to healthy eating. Some students mentioned that a lack of cookware limited food options in terms of what foods could be prepared. Cold storage for foods were often limited, and storage and shelf life were factors students considered when purchasing food. These factors impacted student food choices: “*I used to eat healthy, not like that much healthier, but I would eat more fruits and vegetables, but now I can’t really like keep them in my room.”*

#### Location

The location of homes, dorms, and campus in relation to grocery stores was cited as a barrier. A student who lived in the dorms described lack of accessibility of fresh foods by comparing the dorm offerings to *“a gas station market.”* Aside from distance, students mentioned that transportation, arm-carrying capacity, and ease of transport were factors that limited their food choices. One student who studied abroad mentioned that location was a barrier she recognized to a greater degree upon her return to the US with regard to the city environment*: “in Europe everything was walking distance. I’d just go around the corner and boop! Grocery store, very convenient. [Here,] its America you need a car. [laughter] There’s no way around it - everything is so far away.”*

### Barrier – Macrosystem

#### Cost

Cost was the most frequently mentioned barrier to healthy eating. Almost all students identified money as a barrier to healthy eating, because the cheapest foods often tend to be unhealthy. Students who lived in the dorms noticed the price disparity in *“a little thing of strawberries is $10”* compared to *“musubis* [slice of spam on top of rice wrapped with seaweed] *at $1,”* identifying not only the cost of food in Hawai’i but also the cost of fresh fruit as a barrier in Hawai’i. Cost influenced student food choices in purchasing food.

For students who were beginners in learning to budget, food cost and expenses were a large adjustment, especially after moving from home or not eating on a meal plan anymore. One student described the surprise at the challenge of budgeting: *“all of a sudden the food money comes out of your pocket, that’s like a big dent in your wallet.”*

Other students mentioned that cost was also complicated by other logistical considerations such as travel expenses, gas money, and coordinating with other students to buy food products in bulk.

Students from out of state also reported experiencing “*sticker shock*” because most students are used to comparatively cheaper prices in their hometowns.

#### Lack of education

Students also reported feeling unprepared to make informed food choices upon entering into college. While some students mentioned that they had life skills classes or health courses, they noted a lack of nutrition education in the high school curriculum. *“Nutritional education, I think should be more pushed. Maybe in high school [….] if not in junior high. Like when I went to junior high, we had one class, where it was more like sewing and cooking but you didn’t really learn nutrition that much. And then, I don’t remember learning any nutrition in high school. I think it would be a good requirement to put some kind of nutrition education in schools, more than it is [now].”*

### Enabler – Individual

#### Knowledge

Nutrition knowledge was identified as a key factor in promoting healthy eating: *“There was a book I read called “In Defense of Food” and I feel like after reading that, I’ve like totally changed my whole perspective on eating as well as the way I’ve looked at food [….] it really changed the way I buy food and how I eat food and how I make food.”* Some students identified key books or documentaries which challenged them to think about their eating habits, which they had previously never considered before.

#### Attitudes and beliefs

Some students reported core beliefs related to food that enabled them to eat healthier, like *“You feel better when you eat healthier”* or “*when you go and eat food, it’s kind of like a mental thing - you have the physical refresh of the food, the calories but you also have the time to relax and think”*. By taking time to eat healthy food, students reported that they felt better, which became an important consideration when they choose foods.

#### Attitudes and beliefs: Prioritization

Prioritization of healthy eating was more important to some students than others. Healthy eating was a priority for some for optimal functioning of the body. *“I’m an athlete though so it’s really important in my life to feed myself right so I can perform at my best or practice at my best. I know when I don’t eat good, I can tell my practices aren’t as good. For me just eating healthy or eating the right things to fuel my body is a priority.”*

#### Attitudes and beliefs: Thriftiness

Students sought to avoid food waste and overspending by preparing no more than they could eat or store given their budget and facilities. By eating and preparing vegetables instead of purchasing a meal from the food court, students noted that they spent significantly less and were able to get their money’s worth. One student reported a sizable price difference between packing a salad lunch for two instead of buying from the food court for lunch: *“We could go pick up a head of lettuce and it would feed us for half a week and it was $2 -$3 so instead of paying $15 to […] to the food court. We could have six [meals] for a week for significantly less.”*

#### Dietary restrictions

Several students with diet-related conditions, such as a gluten allergy or lactose intolerance, mentioned that this motivated them to eat healthfully. Their nutrition knowledge had to increase due to their dietary restriction, and they were forced to change their way of eating in order to avoid malaise. *“When I moved here I ate really unhealthy and then I was getting really sick[….] it was gluten, and if I could eat, it I would eat it everyday[….] but I stopped eating that [….] but to do that I did really watch what I eat.”*

#### Behaviors

Some students reported certain behaviors as a motivator for eating healthy, such as meal preparation or exercising. “*When I go exercise, since I’m exercising healthy I’m going to start eating healthy and it kind of starts this cycle for me.”* For some students, healthy eating for improved workouts, or eating healthy to avoid undoing the effort done in the workout were a significant enabler of healthy eating.

### Enabler – Social environmental

#### Parental influence

Some students stated that eating healthy foods was easier in the home environment where there were other people partially accountable for their eating behaviors. For some students, eating behaviors did not change much upon entering college even with the physical environment changed, because they felt that *“the thing that impacts my life the most about my healthy eating choices, exercise choices, [and] lifestyle is what started a long time before college: It’s how I was raised. It’s what my parents fed me, what they thought was important […] that also impacted what I think now as well.”*

#### Peer support

Peer support was identified as a key enabler of healthy eating habits, especially though modeling behavior, providing encouragement, or introducing new tips. *“To have that friend to enable [get you to try and] change your lifestyle habits, it’s a huge [...] plus.”*

### Enabler – Physical environmental

#### Institutional environment

While the institutional environment was identified as a barrier for some students, other students praised the campus offerings like the farmer’s market, the free garden spaces on campus organized by the student organic farming training club, or the cafeteria consistently providing fruits, vegetables, and options for a balanced meal. *“[At] UH I think its pretty good - you could get a salad every day and there are fresh fruits all over the place and brown rice.”*

#### Living situation

The living situation of students influenced the food environments for students. For some students, the transition from home life to college life improved healthy eating by improving access to healthy foods. *“My parents worked as hard as they could. […] We always had food on the table but it was not always the best food. For me, going to college and seeing all of the healthy food was kind of my kick start to be like, ‘alright, I’m gonna go to the gym, I’m gonna go eat salads for lunch, I’m gonna try and be healthier with my lifestyle’ […] that was a big factor for me […] actually having the accessibility to all of this stuff.”*

### Enabler - macrosystem

#### Social media

Although commercials and advertising for foods do not necessarily display healthy foods, students reported learning more about healthy food through social media. Some students reported following certain health bloggers to find inspiration, which some noted is becoming “trendy.”

#### Cost

Students mentioned money as an enabler for healthy eating. With more funds, students had more options, including more health foods. Students noted that while fresh foods typically cost less than prepared foods, food is more expensive in Hawai’i than other places in the US. With a lower cost of living, students reported eating healthier in other geographical locations than in Hawai’i: *“I lived in Washington so fresh food there is a lot cheaper and a lot easier to access than here and so I ate super healthy every day.”*

## Discussion

This study revealed barriers and enablers of healthy eating in college students in Hawai’i at the four levels of influence in the socio-ecological model. Findings may be used to inform additional research in this population or interventions targeting college students in Hawai’i.

Knowledge served as both a key barrier and key enabler on the individual level. This finding is in concordance with previous studies that have identified knowledge as an enabler of healthy eating in college students [11, 17]. To address the lack of knowledge some students described, one possibility is offering a health class to improve nutrition education. This suggestion has been offered in previous studies to improve eating habits of students [10, 17]. However, college students may have widely differing characteristics and levels of nutrition-related knowledge. More research into tailoring classes to address varying levels of nutrition knowledge, psychosocial characteristics, or health risks may result in more effective targeting of diverse groups of college students [18].

Attitudes and beliefs toward healthy eating in individuals were identified as both a barrier and enabler. As a barrier, some students viewed healthy eating as something that could be postponed, or a lower priority in relation to their school life. Attitudes found in this population were consistent with a previous study demonstrating that those in earlier life stages placed less importance of healthy eating, while those in later life stages deemed healthy eating of greater significance [19].

The perception that healthy food is not convenient food was a common theme. Students often placed healthy food at odds with convenience in stating that healthy food took longer to prepare or required more planning than pre-packaged foods, take-out, or other options. However, convenience food has typically been defined in relation to time and labor required for food preparation; it is not necessarily unhealthy, although preserved or processed foods may have added sugar or salt to preserve flavor [20]. Pre-cut fruits or vegetables and nuts fall within the scope of convenience food definition, and these have been shown to be perceived as healthy snacks by college students [21, 22]. Interventions promoting convenient and healthy food options may be helpful in countering the perception that healthy food is labor-intensive.

At the social environmental level, parental control was viewed as both an enabler and barrier to healthy eating. Students reported that healthy food choices were easier with someone creating a healthier environment for them, or monitoring their behavior. Previous research also indicates that parental influence shapes the child’s perceptions of regularity and normal behavior [23]. Findings of the current study also aligned with previous research regarding the desire to rebel and overindulge in forbidden foods when too much parental control and prohibition is exercised [24].

Aside from family groups, peer groups were also identified at the social environmental level. Part of this peer group includes friends, acquaintances, or a boyfriend. Through friend groups, behaviors are modeled, which can serve as either a barrier or enabler to healthy eating. In the current study’s focus groups, friends were valued as support for making a lifestyle change together especially with encouragement and keeping each other accountable for performing the desired behavior. However, although social support in these focus groups was a reported enabler, other studies in college students have reported peer groups as a barrier by normalizing stress-related eating behaviors, like eating when bored, bingeing on junk food, or eating at irregular times [25]. In the current study, participants mentioned eating when bored as an individual rather than social barrier.

At the physical environmental level, the “all you can eat” style of cafeteria at UHM was deemed both a barrier and enabler of healthy eating, because both healthy and less healthy offerings are provided on an unlimited, regular basis. In order to “get one’s money worth,” some students reported eating beyond satiety. However, with buffet layout, there are ways to reduce mindless overeating or minimize the effect of overeating by portioning food or avoiding more than two different foods on the plate at the same time [26]. Aside from minimizing overeating, placing healthier foods at the front of the cafeteria may increase better food choices in the buffet [27]. A possible intervention could include teaching healthier buffet behavior in conjunction with modifying the physical layout of the buffet.

Location of grocery stores in relation to campus, dorms, or living situation was a barrier for students. A previous study in Hawai’i has been conducted to understand food availability and affordability in local communities based on supermarket or farmer’s market offerings and proximity to bus lines for several communities [28]. Future studies could adapt the aforementioned study’s analysis for food availability and affordability in the UHM college and surrounding neighborhood area by examining rent/housing costs, distance to grocery stores or farmer’s markets, cafeteria offerings, and bus line proximity. It is also recommended that future studies measure travel time to grocery stores from campus as travel time is a convenience-related cost that students consider when making food choices [29].

In examining barriers at all levels, cost was the most frequently mentioned barrier, which aligns with the findings of previous studies [9, 11, 30]. Previous research has revealed that healthy diets cost more on a daily basis than unhealthy diets [31]. Moreover, the cost of living in Honolulu is the highest in the United States [32]. Shipping, importation, distribution, and other factors in Hawaii are components of the high food cost. Limited interventions in the college environment have been conducted with food cost-lowering measures, and none have been done yet in Hawaii. A previous study found that identifying budget-friendly fruit and vegetable options as a point-of-purchase message was effective in increasing fruit and vegetable selection by college students, and future research could determine if this intervention is translatable to Hawaii’s population [30].

Students’ perceived norms played a role in enabling healthy behaviors, as participants perceived health as trendy, especially in social media in the past few years [33]. Some participants regarded social media as a source of health promotion, allowing students to shape their perception of normalized behavior by selecting whom to follow. Students may model behavior seen in social media or use it as reinforcement of healthy behaviors. When healthy behaviors are considered part of social identity, and these attitudes are repeatedly reinforced via social media, students may maintain healthier behaviors through the reinforcing spiral model [34].

### Limitations

The study may have been subject to selection bias, because students who participated in the study knew the topic prior to the focus group and may have had an interest in healthy eating. Half of the students who took part in the study were also from nutrition or health-related majors, which means that their eating habits may not reflect those of the broader student population. Findings may not be generalizable to the rest of the population, nor are the identified barriers or enablers quantifiable.

### Future directions

Future studies could use triangulation to further explore the barriers and enablers through the mix of qualitative and quantitative method research [35]. Aside from further elucidating the barriers and enablers, questionnaires could be administered to quantify or rank barriers or enablers in relation to each other.

Future studies on the physical environment of the school and students’ living conditions would be helpful in determining how to establish a more supportive environment, especially since the physical environment was a commonly identified barrier. Interventions should be focused on addressing the most significant perceived barriers of cost, institution, and location to build a supportive environment for healthy eating.

## Conclusion

This study has identified the perceived barriers and enablers of healthy eating in college students. More barriers than enablers were identified. The largest barriers by socio-ecological level were nutrition knowledge deficit (individual), peer pressure (social environmental), unsupportive institutional environment (physical environmental), and cost (macrosystem). The largest enablers by socio-ecological level were nutrition knowledge (individual), parental influence (social environmental), an institutional environment with consistent healthy offerings (physical environmental), and social media (macrosystem). Results from this study identify potential areas of intervention, such as improving nutrition knowledge (individual), offering more healthy options (physical environmental), or reducing cost of food (macrosystem). However, more research is needed to identify which level of intervention may be most effective in changing food habits, and which barriers or enablers are deciding factors in determining this population’s food choices.

## Additional file

Additional file 1 Question guide for focus groups on barriers and enablers of healthy eating in college students. (DOCX 90 kb)

## Abbreviation

UHM: University of Hawai’i at Mānoa

## Authors’ information

LA is a medical student at the University of Hawai’i John A. Burns School of Medicine. She is a recent graduate from the Department of Human Nutrition, Food and Animal Sciences at the University of Hawai’i at Mānoa. Her research interests are obesity prevention, nutrition interventions, preventive healthcare, and healthcare and food policy.

JB is Associate Professor in the Department of Human Nutrition, Food and Animal Sciences at the University of Hawai’i at Mānoa. She has an interest in obesity prevention and health promotion in diverse populations. Much of her current work centers on nutrition education and development of tools to evaluate nutrition education programs aimed at promoting healthy eating. Her previous work at the University of California, Davis involved the development of two tools, a food behavior checklist and physical activity questionnaire, to be used in the low-income Spanish-speaking community in the U.S. to evaluate nutrition education interventions.

OB is an Assistant Professor in the Department of Public Health at the University of Hawai’i at Mānoa, where she teaches health promotion program design, program evaluation, and the undergraduate capstone seminar in public health. Her research uses a CBPR paradigm, and focuses on household food insecurity, environmental and policy factors affecting dietary behavior in low-income communities, and community-based initiatives aimed at improving healthy food access in low-income areas. She contributed to the development of the environmental measures for the USDA’s SNAP-Ed evaluation framework, and has worked closely with the Hawaii SNAP-Ed program on program planning and program evaluation for more than a decade. Dr. Buchthal is currently working with the Hawaii Department of Health and community agencies on several projects related to mapping and improving healthy food access in Hawaii’s low-income communities.
