# Supplementary material for: Identifying perceived barriers and enablers of healthy eating in college students in Hawai’i: a qualitative study using focus groups
Source: BMC Nutr. 2019 Feb 22;5:16. doi: 10.1186/s40795-019-0280-0 (PMC7050928; doi:10.1186/s40795-019-0280-0)
Supplement: Supplementary file 1 — Question guide for focus groups on barriers and enablers of healthy eating in college students. (DOCX 90 kb) [file 40795_2019_280_MOESM1_ESM.docx]

Opening

Where are you from and what’s your name?

Introduction

Describe healthy eating.

Transition

Thinking of ‘healthy eating in university students’, what comes to your mind?

Think back of the last year(s) being a university student. Did your eating behaviors change since you entered college?

Key

Did your eating behaviors change since you entered college?

Which factors have caused these changes? (or which factors influence current health behaviours)? What barriers and enablers of healthy behavior can you identify?

Which of the previous mentioned factors have had the greatest influence? How and why?

Please tell me a few reasons why students like you might want to eat healthier foods?

Please tell me a few reasons why students might not eat healthy already?

Ending

Do you have any remarks, suggestions, additions?

Soon, we will try to help students make healthier choices. Can you give us some advice on how to promote healthy eating behaviours in students?

Questions adapted from:

1. Ashton LM, Hutchesson MJ, Rollo ME, Morgan PJ, Thompson DI, Collins CE. Young adult males’ motivators and perceived barriers towards eating healthily and being active: a qualitative study. *Int J Behav Nutr Phys Act*. 2015;12:93. doi:10.1186/s12966-015-0257-6.

2. Deliens T, Clarys P, De Bourdeaudhuij I, Deforche B. Determinants of eating behaviour in university students: a qualitative study using focus group discussions. *BMC Public Health*. 2014;14(1):1-22. doi:10.1186/1471-2458-14-53.
